# 4-Chloro-2-[(*E*)-(2-chloro­phen­yl)imino­meth­yl]phenol

**DOI:** 10.1107/S1600536809003924

**Published:** 2009-02-11

**Authors:** Xinli Zhang

**Affiliations:** a Department of Chemistry, Baoji University of Arts and Science, Baoji, Shaanxi 721007, People’s Republic of China

## Abstract

The title compound, C_13_H_9_Cl_2_NO, was crystallized from a methanol solution of 5-chloro­salicylaldehyde and *o*-chloro­aniline. The mol­ecule displays a *trans* configuration with respect to the imine C=N double bond. The N atom is involved in an intra­molecular O—H⋯N hydrogen bond. The two aromatic rings are essentially coplanar, the dihedral angle between them being 7.1 (1)°. A C—H⋯π inter­action is present in the crystal.

## Related literature

For the biological properties of Schiff bases containing O and N atoms, see: Antony *et al.* (1999 ▶); Lumme & Elo (1984 ▶); Yao *et al.* (1999 ▶). For its chemical behaviour, see: Ueno *et al.* (2006 ▶).
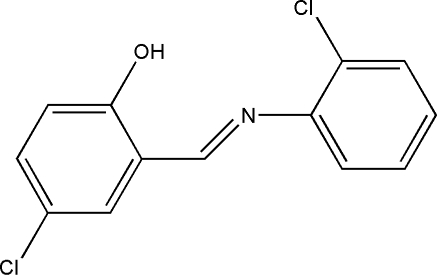


## Experimental

### 

#### Crystal data

C_13_H_9_Cl_2_NO
*M* *_r_* = 266.11
Orthorhombic, 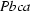

*a* = 7.2693 (13) Å
*b* = 13.0037 (19) Å
*c* = 25.2711 (16) Å
*V* = 2388.8 (6) Å^3^
*Z* = 8
Mo *K*α radiation
μ = 0.52 mm^−1^
*T* = 298 (2) K
0.50 × 0.48 × 0.47 mm

#### Data collection

Siemens SMART CCD area-detector diffractometer
Absorption correction: multi-scan (*SADABS*; Siemens, 1996 ▶) *T*_min_ = 0.780, *T*_max_ = 0.791
11102 measured reflections
2103 independent reflections
1496 reflections with *I* > 2σ(*I*)
*R*_int_ = 0.041

#### Refinement

*R*[*F* ^2^ > 2σ(*F* ^2^)] = 0.037
*wR*(*F* ^2^) = 0.099
*S* = 1.08
2103 reflections
155 parameters
H-atom parameters constrained
Δρ_max_ = 0.20 e Å^−3^
Δρ_min_ = −0.22 e Å^−3^

### 

Data collection: *SMART* (Siemens, 1996 ▶); cell refinement: *SAINT* (Siemens, 1996 ▶); data reduction: *SAINT*; program(s) used to solve structure: *SHELXS97* (Sheldrick, 2008 ▶); program(s) used to refine structure: *SHELXL97* (Sheldrick, 2008 ▶); molecular graphics: *SHELXTL* (Sheldrick, 2008 ▶); software used to prepare material for publication: *SHELXTL*.

## Supplementary Material

Crystal structure: contains datablocks I, global. DOI: 10.1107/S1600536809003924/bq2118sup1.cif

Structure factors: contains datablocks I. DOI: 10.1107/S1600536809003924/bq2118Isup2.hkl

Additional supplementary materials:  crystallographic information; 3D view; checkCIF report

## Figures and Tables

**Table 1:** Hydrogen-bond geometry (Å, °)

*D*—H⋯*A*	*D*—H	H⋯*A*	*D*⋯*A*	*D*—H⋯*A*
O1—H1⋯N1	0.82	1.87	2.603 (3)	147
C11—H11⋯*Cg*1^i^	0.93	2.97	3.549 (3)	122

Symmetry code: (i) 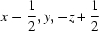. *Cg*1 is the centroid of C8–C13 phenyl ring.

**Fig. 1.**
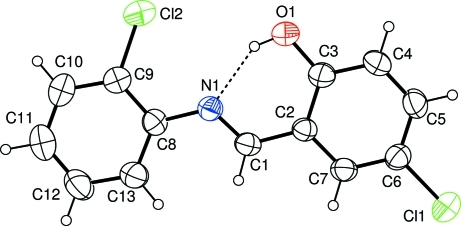
The structure of the title compound with 30% probability ellipsoids. The dashed line represents hydrogen bond.

## supplementary crystallographic information

### Comment

The Schiff base containing some O and N atoms is a new important biological ligand and it shows some interesting biological properties, such as antibacterial, antiphlogistic, anticancer and high catalytic activities (Antony *et al.*, 1999; Lumme & Elo *et al.*, 1984; Yao *et al.*, 1999), so the chemical behavior of the Schiff base has drawn our attention (Ueno *et al.*, 2006). Our research emphasis is focused on the synthesis of the Schiff base. Then, a new crystal structure of the title compound, (I), is reported here.

The molecular structure of (I) are illustrated in Fig. 1. In the structure of (I), the whole molecule is essentially planar with a 7.1 (2)° dihedral angle between the two phenyl rings. The C1═N1 bond distance [1.277 (3)Å] is shorter than the standart 1.28Å value of C═N double bond, indicating a delocalization of π-electron density across the phenyl ring. In addition to the intramolecular O-H..N hydrogen bond, there is also an intermolecular C-H..π interaction (Table 1.)

### Experimental

A solution of 5-chlorosalicylaldehyde (0.1 mmol, 15.7 mg) in methanol (10 ml) was added dropwise to the methanol (10 ml) solution of *o*-chloroaniline (0.1 mmol, 12.8 mg) with stirring. The mixture was stirred at room temperature for one hour and then filtered. After allowing the filtrate to stand in air for 3 d, yellow block-shaped crystals of the title compound were formed in slow evaporation of the solvent. The crystals were collected, washed with methanol and dried in a vacuum desiccator using anhydrous CaCl_2_ (yield 60%).

### Refinement

All H atoms were placed in geometrically idealized positions and constrained to ride on their parent atoms, with C—H distances 0.93Å and *U*_iso_(H) = 1.2 *U*_eq_(C) and O—H distances 0.82Å and *U*_iso_(H) = 1.5 *U*_eq_(O).

### Crystal data

**Table:**

C_13_H_9_Cl_2_NO	*F*(000) = 1088
*M**_r_* = 266.11	*D*_x_ = 1.480 Mg m^−^^3^
Orthorhombic, *P**b**c**a*	Mo *K*α radiation, λ = 0.71073 Å
Hall symbol: -P 2ac 2ab	Cell parameters from 3178 reflections
*a* = 7.2693 (13) Å	θ = 2.9–26.3°
*b* = 13.0037 (19) Å	µ = 0.52 mm^−^^1^
*c* = 25.2711 (16) Å	*T* = 298 K
*V* = 2388.8 (6) Å^3^	Block, yellow
*Z* = 8	0.50 × 0.48 × 0.47 mm

### Data collection

**Table:**

Siemens SMART CCD area-detector diffractometer	2103 independent reflections
Radiation source: fine-focus sealed tube	1496 reflections with *I* > 2σ(*I*)
graphite	*R*_int_ = 0.041
φ and ω scans	θ_max_ = 25.0°, θ_min_ = 1.6°
Absorption correction: multi-scan (*SADABS*; Siemens, 1996)	*h* = −8→8
*T*_min_ = 0.780, *T*_max_ = 0.791	*k* = −15→11
11102 measured reflections	*l* = −30→29

### Refinement

**Table:**

Refinement on *F*^2^	Secondary atom site location: difference Fourier map
Least-squares matrix: full	Hydrogen site location: inferred from neighbouring sites
*R*[*F*^2^ > 2σ(*F*^2^)] = 0.037	H-atom parameters constrained
*wR*(*F*^2^) = 0.099	*w* = 1/[σ^2^(*F*_o_^2^) + (0.0287*P*)^2^ + 1.7853*P*] where *P* = (*F*_o_^2^ + 2*F*_c_^2^)/3
*S* = 1.08	(Δ/σ)_max_ = 0.001
2103 reflections	Δρ_max_ = 0.20 e Å^−^^3^
155 parameters	Δρ_min_ = −0.22 e Å^−^^3^
0 restraints	Extinction correction: *SHELXL97* (Sheldrick, 2008), Fc^*^=kFc[1+0.001xFc^2^λ^3^/sin(2θ)]^-1/4^
Primary atom site location: structure-invariant direct methods	Extinction coefficient: 0.0069 (6)

### Special details

**Table:**

Geometry. All e.s.d.'s (except the e.s.d. in the dihedral angle between two l.s. planes) are estimated using the full covariance matrix. The cell e.s.d.'s are taken into account individually in the estimation of e.s.d.'s in distances, angles and torsion angles; correlations between e.s.d.'s in cell parameters are only used when they are defined by crystal symmetry. An approximate (isotropic) treatment of cell e.s.d.'s is used for estimating e.s.d.'s involving l.s. planes.
Refinement. Refinement of *F*^2^ against ALL reflections. The weighted *R*-factor *wR* and goodness of fit *S* are based on *F*^2^, conventional *R*-factors *R* are based on *F*, with *F* set to zero for negative *F*^2^. The threshold expression of *F*^2^ > σ(*F*^2^) is used only for calculating *R*-factors(gt) *etc*. and is not relevant to the choice of reflections for refinement. *R*-factors based on *F*^2^ are statistically about twice as large as those based on *F*, and *R*- factors based on ALL data will be even larger.

### Fractional atomic coordinates and isotropic or equivalent isotropic displacement parameters (Å^2^)

**Table:**

	*x*	*y*	*z*	*U*_iso_*/*U*_eq_	
Cl1	0.07629 (12)	1.13163 (6)	1.12839 (3)	0.0653 (3)	
Cl2	0.41980 (14)	0.79479 (6)	0.83555 (3)	0.0760 (3)	
N1	0.3625 (3)	0.96742 (15)	0.90783 (8)	0.0407 (5)	
O1	0.2932 (4)	0.81908 (14)	0.97404 (8)	0.0734 (7)	
H1	0.3252	0.8457	0.9461	0.110*	
C1	0.3120 (3)	1.03135 (19)	0.94334 (9)	0.0396 (6)	
H1A	0.3132	1.1013	0.9357	0.048*	
C2	0.2530 (3)	0.99774 (18)	0.99498 (9)	0.0366 (6)	
C3	0.2445 (4)	0.89294 (19)	1.00872 (10)	0.0482 (7)	
C4	0.1860 (4)	0.8649 (2)	1.05868 (11)	0.0591 (8)	
H4	0.1806	0.7956	1.0677	0.071*	
C5	0.1357 (4)	0.9376 (2)	1.09521 (11)	0.0519 (7)	
H5	0.0972	0.9178	1.1288	0.062*	
C6	0.1427 (4)	1.04066 (19)	1.08177 (10)	0.0432 (6)	
C7	0.1990 (4)	1.07049 (19)	1.03254 (10)	0.0422 (6)	
H7	0.2014	1.1400	1.0239	0.051*	
C8	0.4229 (3)	0.99867 (19)	0.85743 (9)	0.0393 (6)	
C9	0.4587 (4)	0.9228 (2)	0.82003 (11)	0.0462 (7)	
C10	0.5255 (4)	0.9469 (2)	0.77026 (11)	0.0588 (8)	
H10	0.5487	0.8949	0.7459	0.071*	
C11	0.5574 (4)	1.0476 (3)	0.75699 (12)	0.0627 (8)	
H11	0.6026	1.0641	0.7236	0.075*	
C12	0.5224 (5)	1.1235 (2)	0.79298 (12)	0.0635 (9)	
H12	0.5439	1.1918	0.7839	0.076*	
C13	0.4556 (4)	1.1001 (2)	0.84244 (11)	0.0544 (7)	
H13	0.4319	1.1529	0.8663	0.065*	

### Atomic displacement parameters (Å^2^)

**Table:**

	*U*^11^	*U*^22^	*U*^33^	*U*^12^	*U*^13^	*U*^23^
Cl1	0.0859 (6)	0.0587 (5)	0.0515 (4)	0.0011 (4)	0.0102 (4)	−0.0135 (4)
Cl2	0.1089 (8)	0.0424 (4)	0.0766 (6)	−0.0022 (4)	0.0226 (5)	−0.0113 (4)
N1	0.0478 (13)	0.0375 (12)	0.0367 (11)	−0.0015 (10)	−0.0029 (10)	−0.0003 (10)
O1	0.128 (2)	0.0355 (10)	0.0562 (12)	0.0156 (12)	0.0259 (13)	0.0034 (9)
C1	0.0434 (15)	0.0330 (13)	0.0424 (14)	−0.0013 (11)	−0.0043 (12)	0.0023 (12)
C2	0.0385 (13)	0.0335 (12)	0.0377 (13)	−0.0001 (10)	−0.0036 (12)	0.0011 (11)
C3	0.0614 (18)	0.0379 (14)	0.0452 (15)	0.0094 (13)	0.0001 (14)	0.0028 (12)
C4	0.088 (2)	0.0384 (15)	0.0512 (16)	0.0129 (15)	0.0078 (16)	0.0119 (14)
C5	0.0633 (19)	0.0532 (17)	0.0391 (14)	0.0078 (14)	0.0032 (14)	0.0097 (13)
C6	0.0467 (16)	0.0428 (15)	0.0402 (14)	0.0026 (12)	−0.0029 (12)	−0.0038 (12)
C7	0.0481 (16)	0.0343 (13)	0.0443 (15)	−0.0037 (11)	−0.0030 (12)	−0.0001 (11)
C8	0.0390 (14)	0.0436 (14)	0.0353 (13)	−0.0006 (12)	−0.0059 (11)	0.0024 (11)
C9	0.0472 (17)	0.0435 (15)	0.0479 (15)	0.0012 (12)	−0.0004 (13)	−0.0024 (12)
C10	0.060 (2)	0.068 (2)	0.0478 (16)	0.0039 (16)	0.0083 (15)	−0.0077 (15)
C11	0.063 (2)	0.080 (2)	0.0456 (16)	−0.0032 (17)	0.0071 (15)	0.0107 (17)
C12	0.082 (2)	0.0572 (18)	0.0518 (17)	−0.0089 (17)	0.0014 (16)	0.0151 (15)
C13	0.073 (2)	0.0429 (15)	0.0475 (16)	−0.0042 (14)	−0.0005 (15)	0.0022 (13)

### Geometric parameters (Å, °)

**Table:**

Cl1—C6	1.738 (3)	C5—H5	0.9300
Cl2—C9	1.733 (3)	C6—C7	1.366 (3)
N1—C1	1.277 (3)	C7—H7	0.9300
N1—C8	1.407 (3)	C8—C9	1.391 (4)
O1—C3	1.347 (3)	C8—C13	1.393 (4)
O1—H1	0.8200	C9—C10	1.384 (4)
C1—C2	1.441 (3)	C10—C11	1.371 (4)
C1—H1A	0.9300	C10—H10	0.9300
C2—C7	1.397 (3)	C11—C12	1.367 (4)
C2—C3	1.408 (3)	C11—H11	0.9300
C3—C4	1.381 (4)	C12—C13	1.375 (4)
C4—C5	1.371 (4)	C12—H12	0.9300
C4—H4	0.9300	C13—H13	0.9300
C5—C6	1.383 (4)		
			
C1—N1—C8	122.5 (2)	C6—C7—H7	119.6
C3—O1—H1	109.5	C2—C7—H7	119.6
N1—C1—C2	121.6 (2)	C9—C8—C13	117.1 (2)
N1—C1—H1A	119.2	C9—C8—N1	117.9 (2)
C2—C1—H1A	119.2	C13—C8—N1	124.9 (2)
C7—C2—C3	118.4 (2)	C10—C9—C8	121.5 (3)
C7—C2—C1	119.6 (2)	C10—C9—Cl2	118.7 (2)
C3—C2—C1	122.0 (2)	C8—C9—Cl2	119.8 (2)
O1—C3—C4	119.2 (2)	C11—C10—C9	119.9 (3)
O1—C3—C2	121.2 (2)	C11—C10—H10	120.1
C4—C3—C2	119.6 (2)	C9—C10—H10	120.1
C5—C4—C3	121.0 (3)	C12—C11—C10	119.7 (3)
C5—C4—H4	119.5	C12—C11—H11	120.1
C3—C4—H4	119.5	C10—C11—H11	120.1
C4—C5—C6	119.5 (3)	C11—C12—C13	120.7 (3)
C4—C5—H5	120.2	C11—C12—H12	119.6
C6—C5—H5	120.2	C13—C12—H12	119.6
C7—C6—C5	120.6 (2)	C12—C13—C8	121.2 (3)
C7—C6—Cl1	120.5 (2)	C12—C13—H13	119.4
C5—C6—Cl1	118.9 (2)	C8—C13—H13	119.4
C6—C7—C2	120.7 (2)		
			
C8—N1—C1—C2	−179.1 (2)	C1—C2—C7—C6	−179.9 (2)
N1—C1—C2—C7	180.0 (2)	C1—N1—C8—C9	−174.2 (2)
N1—C1—C2—C3	−1.2 (4)	C1—N1—C8—C13	8.1 (4)
C7—C2—C3—O1	179.4 (3)	C13—C8—C9—C10	0.6 (4)
C1—C2—C3—O1	0.6 (4)	N1—C8—C9—C10	−177.4 (3)
C7—C2—C3—C4	−0.8 (4)	C13—C8—C9—Cl2	−179.5 (2)
C1—C2—C3—C4	−179.6 (3)	N1—C8—C9—Cl2	2.5 (3)
O1—C3—C4—C5	179.8 (3)	C8—C9—C10—C11	−0.1 (4)
C2—C3—C4—C5	0.1 (5)	Cl2—C9—C10—C11	180.0 (2)
C3—C4—C5—C6	0.3 (5)	C9—C10—C11—C12	−0.2 (5)
C4—C5—C6—C7	0.1 (4)	C10—C11—C12—C13	0.1 (5)
C4—C5—C6—Cl1	179.4 (2)	C11—C12—C13—C8	0.4 (5)
C5—C6—C7—C2	−0.9 (4)	C9—C8—C13—C12	−0.7 (4)
Cl1—C6—C7—C2	179.8 (2)	N1—C8—C13—C12	177.1 (3)
C3—C2—C7—C6	1.2 (4)		

### Hydrogen-bond geometry (Å, °)

**Table:**

*D*—H···*A*	*D*—H	H···*A*	*D*···*A*	*D*—H···*A*
O1—H1···N1	0.82	1.87	2.603 (3)	147
C11—H11···Cg1^i^	0.93	2.97	3.549 (3)	122

Symmetry codes: (i) *x*−1/2, *y*, −*z*+1/2.
